# Assessment of a novel multi-array normalization method based on spike-in control probes suitable for microRNA datasets with global decreases in expression

**DOI:** 10.1186/1756-0500-7-302

**Published:** 2014-05-17

**Authors:** Alain Sewer, Sylvain Gubian, Ulrike Kogel, Emilija Veljkovic, Wanjiang Han, Arnd Hengstermann, Manuel C Peitsch, Julia Hoeng

**Affiliations:** 1 Philip Morris International Research & Development, Philip Morris Products S.A., Quai Jeanrenaud 5, 2000 Neuchâtel, Switzerland; 2 Philip Morris International Research & Development, Philip Morris Research Laboratories GmbH, Fuggerstrasse 3, 51149 Cologne, Germany

**Keywords:** MicroRNA, Microarray, Spike-in controls, Normalization, Differential expression, Data quality metrics

## Abstract

**Background:**

High-quality expression data are required to investigate the biological effects of microRNAs (miRNAs). The goal of this study was, first, to assess the quality of miRNA expression data based on microarray technologies and, second, to consolidate it by applying a novel normalization method. Indeed, because of significant differences in platform designs, miRNA raw data cannot be normalized blindly with standard methods developed for gene expression. This fundamental observation motivated the development of a novel multi-array normalization method based on controllable assumptions, which uses the spike-in control probes to adjust the measured intensities across arrays.

**Results:**

Raw expression data were obtained with the Exiqon dual-channel miRCURY LNA™ platform in the “common reference design” and processed as “pseudo-single-channel”. They were used to apply several quality metrics based on the coefficient of variation and to test the novel spike-in controls based normalization method. Most of the considerations presented here could be applied to raw data obtained with other platforms. To assess the normalization method, it was compared with 13 other available approaches from both data quality and biological outcome perspectives. The results showed that the novel multi-array normalization method reduced the data variability in the most consistent way. Further, the reliability of the obtained differential expression values was confirmed based on a quantitative reverse transcription–polymerase chain reaction experiment performed for a subset of miRNAs. The results reported here support the applicability of the novel normalization method, in particular to datasets that display global decreases in miRNA expression similarly to the cigarette smoke-exposed mouse lung dataset considered in this study.

**Conclusions:**

Quality metrics to assess between-array variability were used to confirm that the novel spike-in controls based normalization method provided high-quality miRNA expression data suitable for reliable downstream analysis. The multi-array miRNA raw data normalization method was implemented in an *R* software package called *ExiMiR* and deposited in the Bioconductor repository.

## Background

MicroRNAs (miRNAs) are short (on average 22 nucleotides long) non-coding RNA molecules that are expressed in almost all eukaryotes. They post-transcriptionally repress the expression of their target genes using the cellular RNA interference mechanism [1]. The latest release (Release 20: June 2013) of the miRBase database contains 1,872 and 1,186 precursor miRNAs for human and mouse, respectively [2]. Although this number will grow because of advances in sequencing technologies, it will almost certainly remain significantly lower than the estimated number of protein-coding genes, which is currently approximately 20,000 in both these species [3]. This difference between the numbers of miRNA and protein-coding genes is a major reason for the broad interest in these small regulatory miRNAs: they can be used as biological process markers that are easier to study than the ten times more numerous messenger RNAs (mRNAs) generated by transcriptomics [4]. Furthermore, many functional studies have revealed that miRNAs play crucial roles in fundamental biological processes, particularly in disease-related processes such as cancer [5,6]. These two factors explain the growing interest and importance of miRNA research and the wide perspectives it offers for the near future.

Expression profiling frequently serves as the basis of investigations involving miRNAs. In recent years, high-density oligonucleotide arrays have become the most popular profiling technology because they allow the simultaneous quantification of hundreds of miRNAs, similar to the way mRNAs are quantified in transcriptomics. This technology is currently less expensive than high-throughput sequencing and faster than quantitative reverse transcription–polymerase chain reaction (RT-qPCR); however, it is also noisier than RT-qPCR because of the high-throughput approach [7]. Commercial miRNA arrays are available, and comparative studies have revealed considerable inter-platform discrepancies among them [8-11]. The dual-channel Exiqon miRCURY LNA™, which was included in these studies, was reported to yield good-quality results. Affymetrix also has miRNA versions of its GeneChip® arrays, which have the advantage of being genuinely single-channel platforms.

The raw data generated by all of these miRNA profiling platforms must be normalized before the results from any single hybridized array can be compared with the results from another. The normalization step involves correcting for the experimentally unavoidable biases associated with each hybridized array, which would otherwise compromise the reliability of the subsequent comparisons. Several computational approaches to address this task have been developed. These approaches differ in various aspects such as the signal intensity dependence of the correction, or the single- or multiple-array design [12-14]. In recent years, one approach has been to apply normalization algorithms developed for transcriptomics directly to miRNA raw data, for example, LOWESS or quantile normalization [15]. As pointed out recently [16,17], this approach is questionable because of fundamental differences between the miRNA and mRNA arrays. Indeed, the number of probes on a miRNA array is significantly smaller than the number of probes on an mRNA array; typically 46,228 probes on the GeneChip® miRNA and 1,300,000 probes on the U133 Plus 2.0 Affymetrix array. Furthermore, the 22-nucleotide-long mature miRNAs are hybridized directly and integrally on the array, whereas mRNAs are probed in several loci distributed over their full length, which might span several kilobases. Because of these differences, the summarization step [18] becomes merely a “within-array” technical replication in the case of miRNAs. It is important, therefore, to give due consideration to these features when normalizing miRNA raw data.

Because of the challenges associated with the normalization of raw data from miRNA arrays, particularly when there is a global decrease in expression, a limited number of satisfactory approaches have been proposed [12,16,17,19]. The basic principles and procedures of these approaches are very similar. First, calibration quantities are extracted from each array and compared between arrays. The raw data from each array are then corrected to ensure optimal alignment of the calibration quantities across all arrays. In their study [16], Sarkar *et al.* used the signal intensities of the control probes that targeted the spike-in RNA as the calibration quantities. These molecules constitute an ideal calibration means because they are designed to exhibit the same concentration in all the samples that are hybridized on the arrays. A further feature is the hybridization strategy of the RNA samples, which follows the single- or dual-channel design inherent in the profiling technology. The dual-channel Exiqon platform that was used in the present study can lead to complications in the case of multi-factorial experimental designs. A simplifying strategy is the “common reference design”, which hybridizes the same reference sample on the first channel across the whole experiment and, at the same time, hybridizes the actual samples on the second channel. In this case, a “pseudo-single-channel” normalization approach is possible, as it has already been tested for mRNA array data [20]. This strategy, however, has never been assessed explicitly for miRNAs by comparing it with genuinely single-channel data.

The above considerations show that some fundamental issues still exist in the preprocessing of miRNA expression data. To obtain high-quality, reliable miRNA expression values for the investigation of relevant biological processes, appropriate normalization methods need to be developed, tested, and applied. The results of the present study contribute to addressing these issues in the following ways:

● First, a novel multi-array normalization method was developed, which uses the intensities of spike-in control probes and is based on a set of controllable assumptions. The method is an alternative multi-array and intensity-dependent correcting procedure to the one proposed by Sarkar *et al.*[16]. Here, “multi-array” means that the normalization corrections for a given array are calculated from the intensities of the spike-in control probes in all the arrays contained in the experimental data. The normalization method was applied to raw data obtained with the Exiqon miRCURY LNA™ platform, but it can also be used with other platforms as long as suitable spike-in controls are available.

● Second, multiple pipelines for generating normalized miRNA expression data were run on the same raw data. To assess the “pseudo-single-channel” hybridization strategy used with the dual-channel Exiqon platform, single-channel Affymetrix raw data were also considered. The results of these preprocessing pipelines were compared, which revealed useful information on the performance of each of their components.

● Third, a dataset generated in an experiment with a quantitative treatment versus control design was used to measure explicitly the various contributions to the variability in the miRNA expression data. This allowed us to develop several quality control metrics based on the coefficient of variation (CV), which were used to compare the pipelines.

● Fourth, the differential expression of the miRNAs was computed for all the pipelines and compared with the corresponding RT-qPCR values for a representative miRNA subset. The results suggested that the considered dataset contained a global decrease of miRNA expression and that the spike-in controls based normalization (SCN) method was appropriate in this situation.

Together, the results showed that the Exiqon platform provided high-quality “pseudo-single-channel” raw data and that the novel normalization method based on spike-in controls globally produced the most consistent normalized data. The SCN method was implemented in an *R* software package called *ExiMiR* that accepts raw data formats obtained with the widely used bioinformatics packages *limma* and *affy*. *ExiMiR* has been deposited in the Bioconductor repository [21].

## Results

### Assessment strategy for the spike-in controls based normalization method

To assess the novel SCN method, a careful analysis of the quality of miRNA expression data obtained with the Exiqon miRCURY LNA™ microarray technology was performed. Lung and blood samples were taken from a mouse cigarette smoke inhalation experiment and processed according to the preprocessing pipelines described in Additional file 1: Table S1. The pipelines include the generation of raw expression data as well as the subsequent normalization procedure. Raw data obtained on the single-channel Affymetrix platform were used as benchmarks for the raw data obtained on the dual-channel Exiqon platform using the “common reference design” data and the resulting “pseudo-single-channel” hybridization strategy (see “Methods” for details of the design and Additional file 2 “Supplementary Results” for a comparison of the raw data and detection calls obtained on the Exiqon and Affymetrix platforms). To take the specificity of miRNA arrays into account, the SCN method was applied and compared with a collection of other methods, such as the LOWESS and quantile normalizations (see Addtional file 1: Table S1). An alternative method based on spike-in controls was also included (SCVSN). It was chosen as the representative of several closely related approaches [19,22,23], all of which use spike-in controls and are expected to differ only in a few specific aspects, as turned out to be the case for SCN and SCVSN. The final outcome of the expression data processing generated the miRNA differential expressions induced by the smoke treatment. The values obtained using the various pipelines in Addtional file 1: Table S1 were compared with one another and with RT-qPCR measurements. To identify the factors that affected the final results, several data quality control metrics were introduced during the intermediate steps of processing.

### Main elements of the spike-in controls based normalization method

The SCN method was developed to address the specificities of miRNA arrays compared with mRNA arrays, as mentioned in the “Background”. As explained in detail in “Methods”, SCN uses a multi-array approach and spike-in control probes to construct an intensity-dependent normalization correction function (*ΔE*). It is based on the idea that in the absence of sample-specific biases, the intensities measured by a given spike-in control probe in one array will be very close to the intensities measured by the same spike-in control probe in the other arrays, because the concentrations of the corresponding spiked-in RNA molecules are the same for all the samples. As a consequence, the measured intensities of the spike-in control probes allow the sample-specific biases to be quantified. The results can be then used to calculate the corrections that should be applied to the intensities measured by the miRNA probes to remove the sample-specific biases, which is precisely the goal of the raw data normalization.

The validity of the SCN method relies on four assumptions, labeled A0–A3:

A0: The deviations that affect the spike-in control probes are the same as the deviations that affect the miRNA probes.

A1: A substantial part of the individual variance of a spike-in control probe is “shared” with the other spike-in control probes.

A2: The intensity range covered by the spike-in control probes that map to the same probe set is small enough that the intensity value of the resulting spike-in probe sets is well-defined.

A3: The coverage of the intensity range of all the miRNA probe sets by the intensities of the spike-in control probe sets is sufficient.

As explained in detail in the “Methods” subsection “The spike-in controls based normalization method”, the raw data normalization is performed in two steps. First, the intensity corrections for the spike-in control probe sets are calculated using the “shared” variance guaranteed by assumption A1. As a consequence of this genuinely multi-array approach, the intensity corrections of the spike-in control probe sets display consistency across both spike-in control probe sets and experimental samples. Second, the intensity corrections for the miRNA probe sets are calculated based on the results obtained for the spike-in control probe sets in the first step, as implied by assumption A0. This step includes the construction of a normalization correction function *ΔE*, for which assumptions A2 and A3 are essential. The multi-array and intensity-dependent normalization correction function *ΔE* has to be constructed carefully to ensure its stable extrapolation at high intensities where coverage by the spike-in control probes can be sparse.

### Application of the spike-in controls based normalization method

**Figure 1:**
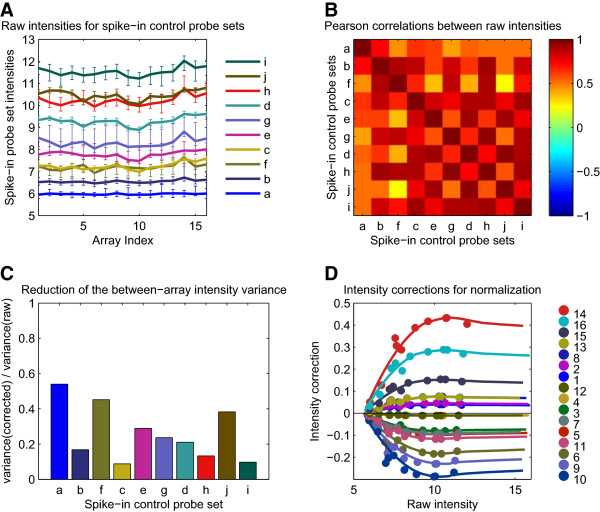
**The spike-in control based normalization (SCN) method. (A)** Plot of the raw intensities for the 10 spike-in control probe sets “*a”*… “*j”* from the 16 arrays of the Exiqon lung dataset. The probe set intensity values were computed as the median values of the corresponding 48 probe intensities and the error bars correspond to the 2.5th–97.5th percentiles of the corresponding distributions. For a given spike-in control probe set, the “coherent deviations” mentioned in the text are estimated by the size of the between-array range of intensity values, divided by the between-array mean intensity value. **(B)** Heat map of the Pearson correlation matrix between all pairs of spike-in control probe sets shown in panel A. **(C)** Ratio between the variance of the raw and normalized intensities for the 10 spike-in control probe sets shown in panels A and B. **(D)** Raw intensity dependence of the spike-in controls based normalization intensity correction function *ΔE* computed for the 16 arrays of the lung dataset. The continuous curves represent the intensity correction function *ΔE(x,k)* defined for the continuous raw intensity values *x* given by the horizontal axis and the 16 discrete array labels *k* depicted in the color legend, while the points correspond to normalization intensity corrections *ΔE*^*(S)*^_*jk*_ for the 10 spike-in control probe sets *j* and the 16 array labels *k* (see the “Spike-in controls based normalization method” section in “Methods”).

**Figure 2:**
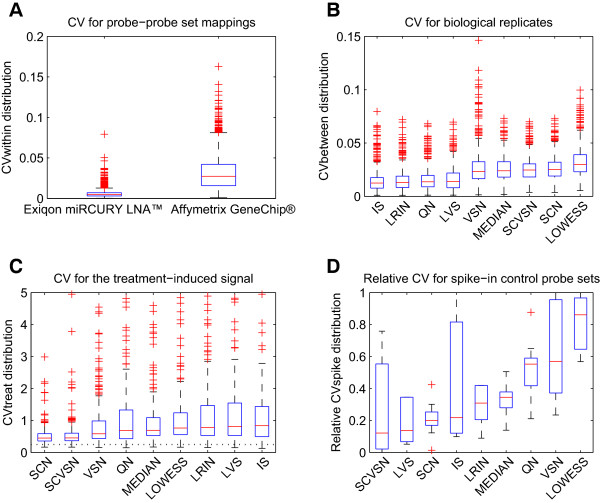
**Quality control metrics using the coefficient of variation (CV). (A)** Boxplot of the CVs between the raw intensity values for the four probes mapping to a given probe set (*CVwithin*), computed for all 595 common mouse miRNA probe sets and for all 16 arrays of the lung dataset, excluding the “absent” detection calls (see Additional file 2 “Supplementary Results”). **(B)** Boxplot of the CVs between the probe set normalized intensity values from the four biological replicates of a given treatment group (*CVbetween*), computed for all 595 common mouse miRNA probe sets and for all four treatment groups of the lung dataset, excluding the “absent” detection calls. **(C)** Boxplot of the ratio between the probe set residual variance and the corresponding modeled treatment response (*CVtreat*), computed for all 595 common mouse miRNA probe sets for the lung dataset, excluding the “absent” detection calls. **(D)** Boxplot of the CVs between the probe set normalized intensity values from all 16 arrays of the lung dataset, computed for the 10 Exiqon spike-in control probe sets. The boxplots for each preprocessing pipeline (described in Additional file 1: Table S1) show the values relative to the range of the corresponding *CVbetween* distribution, which is given by the whiskers in panel B and the interval [0,1] in panel D (*relativeCVspike*, see the “Coefficients of variation” section in “Methods”). Median, red line; first and third quartiles Q1 and Q3, blue box; distribution range given by the two extreme values within the interval [Q1 − 1.5×(Q3 − Q1), Q3 + 1.5×(Q3 − Q1)], black whiskers; outliers, red crosses outside the whiskers.

The successful application of the SCN method to the Exiqon raw lung data processed as “pseudo-single-channel”, which corresponds to the SCN processing pipeline in Additional file 1: Table S1, is illustrated in Figure 1. The intensity curves of the spike-in control probe sets were found to deviate from the expected horizontal lines (Figure 1A), indicating the presence of array-specific biases in the hybridized RNA. The possible origins of these biases are examined in the “Discussion”, together with the related assumption A0. However, as indicated by the high mean value, 0.71, of the Pearson correlations in the heat map (Figure 1B), a large fraction of this variability was “shared” among the spike-in control probe sets “*a*” to “*j*”, as required by assumption A1. The intensity plot in Figure 1A shows that assumptions A2 and A3 were fulfilled for the spike-in control probe sets “*a*” to “*j*” available on the Exiqon miRCURY LNA™ platform. The probe sets “*a*” to “*j*” mapped to well-defined intensity values, and the total intensity range measured on the array was covered sufficiently by the spike-in control probe intensities (see also Figure 1D). The magnitude of the “shared” variance was checked and found to be clearly above the noise level. Indeed, the “coherent deviations” corresponded to approximately 5% of the signal log intensity (Figure 1A), whereas the intrinsic intensity error of the probe sets corresponded to approximately 0.5% of the signal log intensity, as shown by the *CVwithin* values in Figure 2A. Consequently, all the prerequisites were fulfilled for a suitable application of the SCN method to the lung raw data. Following the approach described in “Methods”, the correction function *ΔE* was calculated successfully and its features are displayed in Figure 1C and 1D. Figure 1C shows the variance ratio between the corrected and raw intensities of the spike-in control probe sets. Except for the low-intensity “*a*” and the somewhat noisier “*f*” and “*j*” probe sets, the other probe sets exhibited a substantial variance reduction of at least 70%, suggesting that the curves of the corrected intensities of the spike-in control probe sets will be much flatter than in Figure 1A. The intensity and array dependencies of the reconstructed correction function *ΔE* are shown in Figure 1D. Despite the noisy probe set “*f*” at intensities ~7.5, the *ΔE* curves were smooth and stable and so was the extrapolation at high intensity values (>12). Although other curves might be possible in the high-intensity region by a using slightly different extrapolation parameters (see “Methods”), the expected differences between them and the chosen curve would never exceed 15%. Therefore, the chosen *ΔE* curve is reasonably representative, and can be applied safely to correct the raw intensities of all miRNA probes.

**Figure 3:**
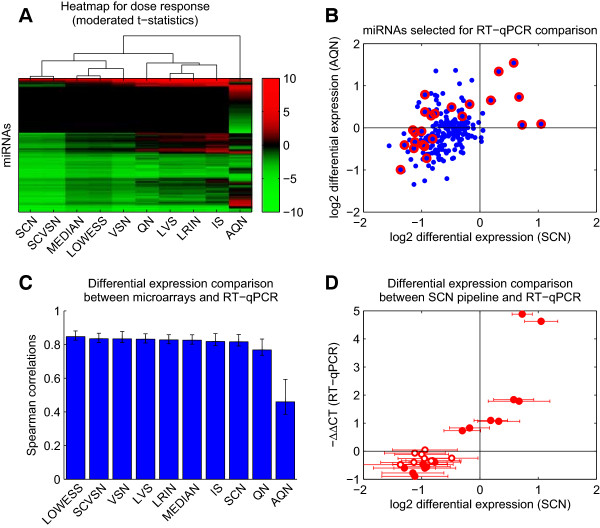
**Differential expression and comparison with RT-qPCR results. (A)** Heat map for the t-statistics obtained from the linear model for the treatment response of the expression values of each miRNA. The dendrogram is based on the Euclidean distance between the t-values obtained from the various preprocessing pipelines (described in Additional file 1: Table S1). **(B)** Scatter plot of the mRNA differential expressions obtained from the normalized data of the SCN vs. AQN preprocessing pipelines. The miRNAs selected for the RT-qPCR experiment are indicated in red. **(C)** Bar chart of the Spearman’s correlation coefficients between the differential expression of the selected miRNAs obtained by RT-qPCR and those obtained from the preprocessing pipelines. The error bars are the 2.5th–97.5th percentiles of the values obtained from a simple leave-one-out re-sampling approach. **(D)** Scatter plot of the differential expressions of the selected miRNAs obtained by RT-qPCR and those obtained from the SCN pipeline. The error bars show the 95% confidence intervals calculated as *t*_0.975,6_ × (SCN differential expression/SCN t-statistic) and the solid circles represent statistically significant -∆∆CT values (*t*-test p-value < 0.05). The horizontal axes of Panels B and D are identical, so that the points of the two plots can be matched.

Because the SCN method proved to be successful, the results were compared with results obtained with a variety of alternative normalization methods using the same raw data. These methods included two approaches that were designed specifically for miRNA expression data (SCVSN and LVS) and more “standard” methods used for gene expression data (see Additional file 1: Table S1). For completeness, quantile normalization was applied to the corresponding Affymetrix raw data (AQN) so that it could be used at the normalized data level (see Additional file 1: Table S1, Figure 3, and Additional file 2: Figures S1, S3, S4, and S5). Note that the Affymetrix raw data were not suitable for use in the SCN method (see Additional file 2: Figure S2).

### Metrics for quality control

For the comparison, all the preprocessing pipelines listed in Additional file 1: Table S1 were considered. Before analyzing the final biological results, several quality control metrics that measure the ratio between the background noise and the exploitable signal at various levels of the experiment based on the coefficient of variation (CV) were first compared (see “Methods”). These metrics allow better identification of the origin of observed differences between the various pipelines in Additional file 1: Table S1. The main results of the various CV computations for the “present” miRNA probe sets for the lung dataset are shown in Figure 2; more exhaustive results are shown in Additional file 2: Figures S3 and S4 for the lung and blood datasets, respectively. The order in which the various normalization methods are displayed in the figures follows the rule of increasing distribution medians with the intention of making it easier to identify similarly behaving normalization methods. *CVwithin* captures the within-array coherence between the four replicated probes that map to each of the 595 common miRNA probe sets on all the 16 arrays (see Additional file 2 “Supplementary Results”). Because low CVs indicate high signal-to-noise ratios, it can be seen clearly in Figure 2A that the Exiqon raw data displayed a very good within-array coherence compared with the corresponding Affymetrix raw data and published results [8]. These findings confirmed the good quality of the Exiqon raw data already observed in the comparisons shown in Additional file 2: Figure S1. *CVbetween* captures the “between-array” coherence between the four biological replicates in each of the four sample groups (“sham”, “low”, “medium”, “high”) in the lung and blood experiments (see “Methods”), thus implying the direct comparison of expression data between different arrays, the results of which strongly depend upon the choice of the raw data normalization methods from Additional file 1: Table S1. It is important to note that even if *CVbetween* has been labeled as a “quality metric”, its lowest values can reflect an artificial reduction of the biological variability, which is not necessarily advantageous for the corresponding normalization methods. The *CVbetween* distributions in Figure 2B show that the nine representative normalization methods applied to the Exiqon raw data can be divided into two groups depending on how efficiently they reduced the biological variability: the “stronger” methods (IS, LRIN, QN, and LVS), and the remaining “moderate” methods (VSN, MEDIAN, SCVSN, SCN, and LOWESS). The SCN and closely related SCVSN methods described in the previous section belonged to the “moderate” methods group. Both these methods are based on the spike-in controls, but SCVSN uses the variance stabilization method to compute the correction function *ΔE*[16,25]. *CVtreat* measures the magnitude of the treatment-induced signal with respect to the background noise, which is defined by the residual intensity values obtained after the subtraction of the treatment-induced signal (see “Methods”). *CVtreat* constitutes an extension of *CVbetween* to differentially treated samples and is also strongly dependent on the normalization method. Because it corresponds roughly to the inverse of a *t* statistic, *CVtreat* provides similar information as the number of differentially expressed miRNAs. However, it is not a measure of sensitivity because the RT-qPCR measurements are not used for comparison. Figure 2C shows that the *CVtreat* results were quite different from the *CVbetween* results. The SCN and SCVSN approaches clearly outperformed all the other approaches in terms of *CVtreat*, in particular the members of the “stronger” methods group as shown in Figure 2B. Although the preprocessing pipelines exhibited global signals with variable strengths, the 5th percentiles of all of them were very close; all were situated below 0.25. This finding suggests that the miRNAs with the strongest differential expressions were very likely to have been captured equally well by all approaches (see Figure 3 for more details). The results obtained up to this point show that the SCN and SCVSN approaches performed well: they were the best when measured by *CVtreat* and intermediate when measured by *CVbetween*, which is exactly the inverse of the quantile normalization (QN) method. To further characterize the performances of the normalization methods applied to the Exiqon raw data, *relativeCVspike* was computed to quantify the between-array noise of the spike-in control probes and to compare it with the between-array noise of all probes, as captured by *CVbetween* and shown in Figure 2B (see “Methods”). Because *CVbetween* tests biological replication on miRNA probes and *relativeCVspike* tests technical replication on spike-in control probes, the distribution of *relativeCVspike* is expected to extend preferentially over the lower part of the [0,1] interval. Because SCN and SCVSN both use the intensities of spike-in control probes to perform the raw data normalization, *relativeCVspike* is expected to be more advantageous for them. Figure 2D shows that the interquartile ranges (IQRs) of the *relativeCVspike* distributions need to be considered together with the relative medians to describe the results properly. The lowest relative medians of SCVSN, least-variant set (LVS), and invariant set (IS) were indeed accompanied by large IQRs. Inversely, SCN displayed a low relative median and the overall smallest IQR value. Rank-invariant normalization from the LUMI package (LRIN), median normalization (MEDIAN), and QN displayed larger relative medians and intermediate IQR values compared with LVS and IS, the other two members of the “stronger” group (Figure 2B). Variance stabilization normalization (VSN) and LOWESS performed the least well as measured by *relativeCVspike*, with a large IQR value or a relative median close to 1. An interpretation of these results is presented in the “Discussion” section.

### Treatment-induced response and comparison with RT-qPCR results

In addition to the quality control metrics based on the CV, the novel SCN method and the alternative preprocessing pipelines from Additional file 1: Table S1 were assessed based on the specific biological question addressed in the experiments. Here, the aim was to identify miRNAs that were differentially expressed in response to exposure to different doses of cigarette smoke. A linear model approach was used to calculate differential expression of the miRNAs in the samples and a subset of the obtained results was compared with data obtained by RT-qPCR (see “Methods”). Figure 3 and Additional file 2: Figure S5 show the final results for the lung experiment (five-month exposure). The results from the blood experiment (one-day exposure) were not used for the RT-qPCR comparisons, because the treatment-induced signal was rather weak, as can be seen by comparing the results shown in Additional file 1: Figure S3B (lung) and Additional file 2: Figure S4B (blood). Figure 3A shows a heat map for the miRNA dose responses obtained from the nine representative Exiqon-based normalization methods considered in Figure 2 (the comprehensive results are displayed in Additional file 2: Figure S5A). These results are supplemented by the Affymetrix-based AQN pipeline to examine the impact of the normalization step on the differences observed at the raw data level (see Figure 2A and Additional file 2: Figure S1). The dose responses are expressed as the moderated t-statistics obtained for a linear model of the dose-dependent response implemented in the *limma* Bioconductor package (see “Methods”). Overall, the results shown in Figure 3A revealed several groups of similarly behaving preprocessing pipelines, as indicated by the superimposed dendrogram based on the Euclidean distance. This pattern is remarkably consistent with the results presented in Figure 2, because these groups of pipelines agree with the best performing pipelines appearing in Figure 2B and 2C: the four methods that most strongly reduce the biological variability (IS, LRIN, QN, and LVS, referred to as the “stronger” group), and the two spike-in controls based approaches (SCN and SCVSN). Among the “stronger” group, QN was the most distant, essentially as a result of non-shared positive values. SCN and SCVSN yielded the largest number of negatively differentially expressed miRNAs, whereas AQN displayed the most balanced distribution between negative and positive differential expression values. AQN, however, remained far from all the pipelines based on the Exiqon raw data including QN, which also uses quantile normalization. The miRNAs selected for comparison with the RT-qPCR results are shown in Figure 3B on a SCN vs. AQN scatter plot of log2 differential expressions between the “high” and “sham” sample groups. One goal of the RT-qPCR experiment was to confirm the differences between AQN and SCN, the two most “distant” preprocessing pipelines in Figure 3A. As a consequence, all the “intermediate” preprocessing pipelines between AQN and SCN in Figure 3A could be meaningfully included in the comparison. However, because the number of miRNAs measured by RT-qPCR represents only a small fraction of the total number of detected miRNAs, the RT-qPCR results cannot be used to draw definitive conclusions from a comparison of every single preprocessing pipeline with another. Rather, the RT-qPCR results will indicate global similarities between the pipelines, similar to the CV-based metrics shown in Figure 2. A second goal of the RT-qPCR measurements was to confirm (at least partly) the global decrease in miRNA expression that appeared very clearly in Figure 3A for most of the preprocessing pipelines. For these purposes, a subset of 24 miRNAs was selected to best cover the similarities and differences between the differential expression values obtained with SCN and AQN, as shown in Figure 3B. The region (SCN > 0, AQN < 0) was poorly populated so it was not possible to find a reliable representative from this region. For the pairwise comparison between the “high” and “sham” exposure treatment groups (see “Methods”), the log2 differential expressions obtained for 10 representative pipelines from Additional file 1: Table S1 and from the RT-qPCR experiment are presented in Figure 3C. Spearman’s rank correlation coefficient (*r*) was used to quantify the similarity of the values obtained for the 24 selected miRNAs, because this statistic is appropriate when comparing values obtained with different technologies that are related in a non-linear manner. The results supported the global decrease in miRNA expression observed in Figure 3A. Further, the results clearly confirmed the higher quality of the approaches based on the Exiqon raw data with an average *r* value of approximately 0.8 compared with the Affymetrix-based AQN approach, which achieved an *r* value of approximately 0.5. To compare the Exiqon-based pipelines in a meaningful way, the robustness of Spearman’s coefficient was assessed by estimating the 95% confidence intervals using a simple leave-one-out re-sampling approach. The results confirmed the results that QN was slightly separate from the other Exiqon-based approaches (Figure 3A), which were, in turn, difficult to order in a definitive manner, as indicated by the error bars resulting from the moderate size of the subset of 24 miRNAs selected for the RT-qPCR measurements (Figure 3C). The performance of the SCN method is shown in a scatter plot of the “high” vs. “sham” log2 differential expressions obtained with the SCN pipeline and from the RT-qPCR measurements (Figure 3D). Overall, the agreement was good, with an *r* value of 0.81 ± 0.04. Differences in the magnitude of the differential expressions were visible particularly for the positive changes and, as expected, the RT-qPCR values provided a clearer signal than the array-based pipelines listed in Additional file 1: Table S1. For instance, four “intermediate” miRNAs mmu-miR-31/146a/221/342-3p exhibited positive RT-qPCR differential expression values of approximately one, whereas the corresponding values obtained from the SCN pipeline were not significantly different from zero, as suggested by the error bars showing the 95% confidence intervals (Figure 3D). In the lower left corner of Figure 3D, the global negative changes observed in both the microarray data and in the RT-qPCR results can be seen, even though some the RT-qPCR differential expression values were not statistically significant when considered on an individual basis.

## Discussion

The well-designed experimental set-up on which this study was based included a quantitative single-factor treatment, sample groups of biological quadruplicates, and high-quality spike-in controls on the microarray. This set-up achieved results that were sufficiently robust from a statistical point of view, thereby substantiating the conclusions. The novel SCN method and the quantitative comparisons of the miRNA expression profiling pipelines are discussed below.

### The spike-in controls based normalization method

The normalization of raw microarray data is essential to allow unbiased comparisons between different arrays. Usually, features that are invariant across all the arrays are identified and used to calibrate each individual microarray. For instance, quantile normalization methods such as robust multi-array (RMA) and GC content RMA (GCRMA), which are widely used for gene expression analysis, assume that the distribution of the probe intensities is the same across all the arrays. The normalized intensity values for each probe are then obtained using this common distribution. For miRNA arrays, other invariant features have been used to normalize the data − for example, the “nonchanging” or “least-variant” probes [12,17]. In the present study, the spike-in control probes available on the Exiqon miRCURY LNA™ arrays were employed to derive an intensity- and array-dependent correction function *ΔE* that was used to correct the raw intensity values, as recently attempted using the variance stabilization method [16,25]. A similar spike-in controls based strategy has been developed successfully for low-density gene expression arrays [28], which, like miRNA arrays, contain a relatively “small” number of array probes, as explained in the “Background”.

#### Array-specific biases

Various types of array-specific biases that can affect the raw data should be considered before examining the validity of the four assumptions A0–A3 that were applied in developing the SCN approach. The biases can be grouped under the following three categories based on their source:

1. Biases that originate from the “biological” RNA, which affect only the miRNA probes. These biases arise from both biological variability, as observed in biologically replicated samples, and treatment-induced effects, as observed in differentially treated samples.

2. Biases that originate from the “synthetic” spiked-in RNA, which affect only the spike-in control probes.

3. Biases that appear only after the “biological” and “synthetic” RNAs are mixed, which affect both the spike-in control probes and the miRNA probes.

Simply stated, assumption A0 affirms that, in the context of normalization, the effect of bias (3) dominates the effects of biases (1) and (2), which indicates that the correction function *ΔE* from Figure 1D can be used to adequately normalize the miRNA probes. The detailed reasoning is as follows: as a consequence of A0, the array-specific biases observed on the spike-in control probes (Figure 1A) are assumed to reflect mainly the effect of bias (3). Hence, the correction function *ΔE* (shown in Figure 1D), which is based on these deviations in the spike-in control probes, can be applied to the miRNA probes to remove the effect of bias (3). Because the biological variability from the effect of bias (1) can be disregarded as a consequence of A0, it is expected that *ΔE* will reliably correct the main array-specific biases on the miRNA probes, while preserving the treatment-induced variability contained in the effect of bias (1).

#### Validity of assumption A0

That biological variability from the effect of bias (1) could be disregarded was confirmed *a posteriori* by the results shown in Figure 2B. As measured by *CVbetween*, the variability in probe set intensities observed over biological replicates with the SCN approach was similar to the variability in intensities with alternative approaches, except for the methods (IS, LRIN, QN, and LVS) that formed the “stronger” group, which, however, performed less well on the other metrics (see Figure 2C and 2D). Thus, neglecting the biological variability from the effect of bias (1) did not seem to be a significant problem in this dataset. However, it should be noted that this finding may not hold true for other datasets. It is, therefore, crucial that the underlying experiments are carried out in a controlled manner, as was the case here. The same requirement also applies to the generation of the synthetic spiked-in RNAs. The manufacturer (Exiqon) explicitly guaranteed that this was indeed the case; therefore, the effect of bias (2) could be also regarded as minor compared with the effect of bias (3). Consequently, assumption A0 can reasonably be considered as valid in this study, confirming that the deviations from the horizontal lines shown in Figure 1A can be considered a direct measure of the effect of bias (3). Finally, it is useful to note that the technically replicated arrays provide ideal conditions for testing assumption A0 specifically, because the effect of bias (1) will be constant in this case. Further, if effect of bias (2) is considered as minor, then both the spike-in controls and the miRNA probes should reflect the effect of bias (3). Unfortunately, these considerations could not be applied here, because there were no technical replications in the experimental data.

#### Validity of assumptions A1–A3

Assumptions A1–A3 are aimed at the possibility of providing a practical correction for effect of bias (3). Unlike assumption A0, these three assumptions can be verified explicitly based on the raw data. The features tested, such as the concentrations of the synthetic spiked-in RNAs, depend more on the experimental procedures than on the actual array technology. Assumption A1 explicitly tests the coherence of the deviations across intensities and arrays shown in Figure 1A. This assumption is a step further than previous studies, which did not consider the “multi-array” aspect explicitly [16,22,23,28]. Assumption A2 ensures that the signal carried by the spike-in control probes is not excessively noisy, and assumption A3 guarantees that the spiked intensities provide a good coverage of the dynamic range of miRNA expression. When A2 and/or A3 are not fulfilled, the intensity dependence of the correction function *ΔE* illustrated in Figure 1D will be difficult to reconstruct. In such a case, the result will be very similar to an intensity-independent correction, which is almost equivalent to MEDIAN. In this study, A1–A3 were fulfilled in the Exiqon datasets (Figure 1) but not in the Affymetrix datasets (Additional file 2: Figure S2).

The variance ratios in Figure 1C provide a good indicator of the efficiency of the bias correction, on which the SCN method is based. These ratios were already observed to be at least 70%, except for the low-intensity “*a*” and the noisier “*f*” and “*j*” probe sets. The residuals below 30%, as well as the variability among probe sets, can be attributed to uncontrolled factors such as the partial hybridization of the spike-in control probes with the “biological” RNA. The extent of this partial hybridization depends on the specific spike-in control probe sequences and on the variable abundances of the expressed miRNA sequences.

#### Implementation of SCN

Because the validity of the SCN method for the “common reference design” strategy for dual-channel Exiqon arrays has been established, its practical implementation could be addressed. Therefore, for the implementation of SCN, a software tool called *ExiMiR* was developed as an *R* package and deposited in the Bioconductor repository. *ExiMiR* executes the SCN pipeline for input raw data in various formats and compliant with assumption A0, and tests assumptions A1–A3 to ensure the applicability of the method. If the assumptions are not met, the MEDIAN is used instead of SCN, as discussed above. The “pseudo single-channel” preprocessing approach based on the “common reference design” is fully exploited in *ExiMiR*. For example, both raw and normalized data from the dual-channel Exiqon arrays are stored as single-channel “AffyBatch” and “ExpressionSet” *R* objects, so that the data processing is almost the same as for single-channel Affymetrix arrays.

### Comparison of the normalization methods

The outcomes of the SCN preprocessing pipeline were compared with the outcomes from the alternative approaches for normalizing miRNA microarray data that are listed in Additional file 1: Table S1. The results presented in Figures 2 and 3 show that the choice of the intensities of the spike-in control probes as the “invariant features” for the raw data normalization was judicious. The approaches in Additional file 1: Table S1 can also be characterized by the specific “invariant features” used: array-based probe intensity medians for MEDIAN, array-based probe intensity quantiles for QN/AQN, intensity-independent variances for VSN/SCVSN, the Hy5 “common reference” channel for LOWESS, invariant/least-variant set of probes/probe sets for IS/LVS, and the rank of probe set intensities for LRIN. The different invariant features that are used should be considered in interpreting the results of the comparisons between the different methods.

#### Spike-in controls based methods (SCN and SCVSN)

The results of the extensive comparisons between the SCN method and the alternative approaches showed that SCN performed very well, except when measured using *CVbetween*, where it was intermediate (see Figures 2 and 3 and Additional file 2: Figures S3, S4, and S5). First, SCN produced the best combined performance on *relativeCVspike* (third lowest relative median and smallest interquartile range values), in perfect agreement with its design. The *relativeCVspike* distributions of the SCVSN method produced interquartile ranges that were significantly larger than the distributions observed for SCN. The second steps of SCVSN and SCN are very similar (see “Methods” and [16]), which implies that the differences in the *relativeCVspike* distributions arise in their first steps. Consequently, it can be concluded that SCN corrects the intensities of the spike-in control probes better than SCVSN, which further confirms the value of the multi-array approach presented in this work. Second, the SCN results measured by *CVtreat* were better than any of the other approaches tested, which can be explained by the observation that the treatment-induced changes contained in the effect of bias (1) were not altered by the normalization procedure. Therefore, although the RT-qPCR results for the selected differentially expressed miRNAs (Figure 3) showed that the performances of all the approaches except QN (and AQN) were similar, the quality control metrics primarily favored SCN (Figure 2). The only exceptions were the *CVbetween* distributions, where the “stronger” methods (IS, LRIN, QN, and LVS) yielded distributions that were lower overall than the SCN distributions. These results apparently suggest that the other methods produced better results than SCN. This finding is examined in more detail in the next two paragraphs.

#### Methods from the “stronger” group

First, it should be noted that the normalization methods used in the “stronger” methods group generated similar *CVbetween* distributions, and also yielded comparable treatment-induced responses (see Figures 2B and 3A). A closer examination of the “stronger” methods group revealed two categories of approaches: rank-invariant approaches (LRIN and QN) and invariant-set based approaches (IS and LVS). That these two categories manifested themselves with the normalized data is not surprising because these results reflect the algorithmic resemblance within each category. A more intriguing aspect is the observation that the invariant-set based category and the rank-invariant category behaved in similar ways. It might have been expected that the rank-invariant category would resemble the SCN and SCVSN approaches more closely, because these two approaches are based on a set of “invariant” spike-in control probes. A careful examination of the executions of IS and LVS revealed that the invariant sets generated during the calculations were, in fact, much larger than the 10 spike-in controls. Indeed, the invariant sets constituted a significant fraction of the probes measured on the chip; close to 10% (850/9360) of the probes for IS, and 70% (1387/1981) of the probes for LVS. These “dense” sets of invariant probes induced a global realignment of the intensity values across the arrays, which may have caused them to become quite similar to the constraints of the invariant quantiles imposed by the QN method. Thus, the resemblance in the *CVbetween* distributions generated by the invariant-set based category and the rank-invariant based QN method is not very surprising for very large invariant sets.

#### Quantile normalization

Because quantile normalization implemented in QN (and AQN) is used extensively in gene expression analyses, it is worth examining its performance in some detail. The underlying assumption of an invariant distribution of the probe intensities between arrays is not guaranteed to be fulfilled for miRNA arrays, as explained in the “Background”. The effects of this on the results are now examined. First, although quantile normalization preserves the relative ranks of the miRNA differential expression values, their signs were strongly affected, as shown by the results in Figure 3A. Further, these sign changes were not always consistent with the RT-qPCR results (Figure 3C), which decreased the overall performance of the QN methods. Second, QN belonged to the “stronger” methods group that yielded the best results for the variability between biological replicates, as captured by *CVbetween*. However, a comparison of *CVbetween* and *relativeCVspike* (Figure 2D) reveals inconsistencies: many biologically replicated miRNA intensities had a lower CV than the technically replicated spike-in control probe intensities. One consequence of these inconsistencies is the possibility that spike-in control probe sets will be found among the top differentially expressed miRNAs when two sample groups are compared. Furthermore, QN yielded an intermediate treatment-induced signal compared with the other tested pipelines, as captured by *CVtreat* (Figure 2C). These observations indicated that the between-array variability reduction induced by QN was too strong when used with miRNA data. This finding is clearly a consequence of the unfulfilled assumption of identical probe intensity distributions across all arrays, as mentioned above. It should be highlighted, however, that for the limited number of miRNAs with the strongest differential expressions, the QN results might still be correct, as illustrated by the converging positions of the 5th percentile distributions of *CVtreat* (Figure 2C) and by the entirely red top rows of positively differentially expressed miRNAs in the dose–response heat map in Figure 3A.

#### Other normalization methods

The results for the other normalization methods (Figures 2 and 3, and Additional file 2: Figures S3, S4, and S5) are discussed below. MEDIAN consistently performed slightly better than the non-normalizing (NN) approach, showing that MEDIAN, which is closely related to SCN, applies a weak but accurate correction to the raw data. As discussed above in connection with assumptions A2 and A3, the correction function *ΔE* used in SCN can become, at worst, an intensity-independent constant (Figure 1D). If assumption A0 holds, the implication is that deviations of the spike-in controls are the same as deviations of the miRNA probes, then SCN exactly becomes MEDIAN for a constant correction function *ΔE*. Although the variance stabilization normalization (VSN) method uses a more sophisticated non-linear transformation of the raw data, its performance is very similarly to MEDIAN, except when measured by *relativeCVspike*. The low number of measured probes on a miRNA array compared with the number on an mRNA array, may make it difficult for VSN to work efficiently. A similar result was observed for SCVSN, where the variance stabilization produced a broad *relativeCVspike* distribution when applied only to the spike-in controls (Figure 2D). The LUMI-based approaches (LRIN, LRS, and LVSN) displayed comparable performances, which were mainly optimal on *CVbetween* and intermediate on *CVtreat*[27]. As discussed, this was typical of the behavior of the methods in the “stronger” group, which is represented by the quantile normalization approaches. Finally, the effect of including probes that target miRNAs from species other than mouse in the normalization was tested by calculating QNm, where only mouse probes were retained. Although the numerous non-mouse probes generated a different and denser intensity distribution for QN, no significant differences were observed between QN and QNm based on the quality control metrics.

#### Summary

The main challenge for the normalization methods appeared to be in achieving the best balance between a reduction in the between-array variability and optimization of the treatment-induced signal. The SCN method presented in this study demonstrated the highest ability, together with SCVSN, in optimizing the treatment-induced signals. SCN also produced the best distinction between the technical and biological variabilities. Nevertheless, SCN was outperformed by QN and the other similarly behaving methods in terms of reduction of the between-array variability. However, the reduction also implied a transformation of the raw data that strongly affected its internal structure. As a result, some of the features were lost, as was observed for QN and the other similar methods where the treatment-induced signals and the distinction between the technical and biological variabilities were affected. A few methods, for example MEDIAN and VSN, displayed more balanced performances, even though the corrections that were made to the raw data were small. LOWESS was found to be unsuitable for use with a “common reference design”. Clearly, the normalization method should be chosen keeping the above considerations in mind, although the topmost differentially expressed miRNAs are likely to be detected by all the methods tested. However, it is possible that the different specificities of each method might “dilute” these miRNAs among false positives.

### Biological aspects

Although a consideration of the biological aspects of these methods was not the purpose of this study, some aspects of the functional biology revealed by this experiment are discussed briefly. This discussion will help reveal the value of using an appropriate preprocessing pipeline for miRNA array data analysis. It was observed that the exposure of mice to cigarette smoke resulted in a notable dysregulation of miRNA expression in the lung (Figure 3A). The four miRNAs mmu-miR-21/135b/146b/147, which were found to be positively differentially expressed by all the pipelines, albeit with unequal statistical significance, are considered. These miRNAs correspond to the thin red stripe at the top of the heat map in Figure 3A and to the four highest RT-qPCR -*ΔΔCT* values in Figure 3D. mmu-miR-21 has been characterized as an oncogenic miRNA (“oncomiR”) in many tumors, including lung cancer [29]. mmu-miR-147 was found to be activated during the inflammatory response in mice where it functions as a negative regulator of toll-like receptor (TLR)-associated signaling events [30]. A powerful way to determine miRNA function was provided by the mirBridge approach [31], which indicated that mmu-miR-135b and mmu-miR-146b regulate the transforming growth factor-beta (TGF beta) and the NFkB signaling pathways, respectively. Another interesting role for mmu-miR-21 has been suggested [32]: its negative action on the Dicer promoter p63, which might help explain the global decrease in miRNA expression that appeared in response to the smoke treatment (Figure 3A). A similar “miRNAome-wide” phenomenon was reported recently in cancer samples (and also led to the development of a novel normalization method for the last generation of the Affymetrix GeneChip® miRNA arrays) [19]. Therefore, a valuable biological outcome of the present study may be the observation of a global decrease in miRNA expression in a cellular stage that may precede the development of tumors. In the present study, the global decrease in miRNA expression came after a five-month long exposure to cigarette smoke (see “Methods”). The A/J mouse that was used in this study is an animal model for lung cancer, which will develop lung tumors after a long enough exposure, typically 18 months [33].

## Conclusions

A novel multi-array method for normalizing miRNA raw data called the SCN method was assessed in this study. This method uses spike-in control probes to adjust the intensity values across arrays. It is implemented in an *R* software package called *ExiMiR*, which has been deposited in the Bioconductor repository. Because the SCN method is based on four controllable assumptions (A0–A3), it constitutes *a priori* a sounder choice than approaches such as the quantile normalization that were originally developed for gene expression arrays. To characterize its features concretely and to compare it with alternative normalization methods, several quality metrics were introduced to measure the variability of the raw and normalized expression data at different levels (within-array for probes mapping to the same probe set, between-array for biological replication, between treatment groups, and between-array for spike-in control probes). These metrics showed that compared with the other tested approaches, the novel SCN method best preserved the treatment-induced signal and reduced the variability between replicated measurements in a consistent and stable manner. The metrics also led to a deeper understanding of the features and relationships among the tested preprocessing pipelines for miRNA expression data. These findings have provided important information that will help researchers make informed choices regarding the best miRNA preprocessing pipeline to use in a particular study. The array-based results were supported by RT-qPCR measurements, which confirmed the value of the novel SCN method and the suboptimal performance of a quantile normalization approach. The RT-qPCR results also revealed a global decrease of miRNA expression in response to cigarette smoke exposure for which the novel SCN method is an appropriate approach. Further, the biological interpretation of the observed differential expression of four miRNAs revealed the possible relevance of the identified miRNAs.

## Methods

### Sample preparation and hybridization

#### Cigarette smoke exposure

Male A/J mice (Jackson Laboratory Bar Harbor, ME), 9 to 13 weeks of age, were exposed in whole-body exposure chambers to conditioned fresh air (“sham”) or to diluted cigarette mainstream smoke from the reference cigarette 3R4F (University of Kentucky, Lexington, KY) at a target concentration of 75 (“low”), 150 (“medium”), or 300 (“high”) μg total particulate matter (TPM)/liter for 6 hours/day, 5 days/week. For the medium and high groups, exposure was started with a dose adaptation period of 2 hours/day on day 1, and gradually increased to reach the target concentrations by day 17.

These exposure experiments were part of a larger study [33], which was approved in accordance with the Belgium Law on Animal Protection. Animal experiments were performed in an AAALAC-accredited facility [34], where care and use of the mice were in accordance with the American Association for Laboratory Animal Science (AALAS) Policy on the Humane Care and Use of Laboratory Animals (http://www.aalas.org).

#### Sample collection

Blood samples and lung tissue from mice exposed for either 1 day or 5 months were collected between 1 and 3.5 hours after the last exposure or after 24 hours (1 day post-exposure) from four mice per group. Lung tissue was snap frozen in liquid N2 immediately after dissection and stored at −70°C. Blood was collected directly upon dissection using the Mouse RiboPure™-Blood RNA Isolation Kit (Ambion, Austin, TX).

#### RNA preparation

Total RNA (including miRNA) was extracted from frozen left lung lobes and from whole blood using a mirVana™ miRNA Isolation Kit (Ambion, Austin, TX). The quality and quantity of the extracted RNA were analyzed using an Agilent 2100 BioAnalyzer (Agilent Technologies, Inc., Santa Clara, CA) and a NanoDrop ND-1000 (PeqLab, Erlangen, Germany), respectively.

#### Microarray procedures

In this study, the Exiqon (Vedbaek, Denmark) and Affymetrix (High Wycombe, UK) miRNA platforms were used.

#### Exiqon

The experiments using the Exiqon platform were performed by Exiqon (Vedbaek, Denmark). Briefly, total RNA samples (0.75 μg) and reference RNA samples were labeled with Hy3™ and Hy5™ fluorescent labels, respectively, using the miRCURY LNA™ Array power labeling kit (Exiqon), following the procedures described by the manufacturer. No dye swap experiments were performed. Each Hy3™-labeled sample was mixed with the Hy5™-labeled reference RNA sample and then hybridized on the miRCURY LNA™ miRNA Arrays (v.11.0) following the “common reference design” [35]. For each tissue type (lung and blood), the reference RNA consisted in a mixture of RNA extracted from all the samples in the experiment. The miRCURY array contains a number of capture probes for hybridization of a range of synthetic spike-in controls. The spike-in controls “*a”*, “*b”*, “*c”*, “*d”*, “*e”*, “*f”*, “*g”*, “*h”*, “*i*”, and “*j”* were spiked into the labeling reaction at various concentrations. Hybridization was performed according to the miRCURY LNA™ array manual. Following hybridization and processing, the arrays were scanned using the Agilent G2565BA Microarray Scanner System (Agilent Technologies, Inc., Santa Clara, CA) and image analysis was performed using the ImaGene 8.0 software (BioDiscovery, Inc., Santa Clara, CA) to quantify the signals. The raw data were provided by Exiqon as ImaGene text files.

#### Affymetrix

Total RNA (1 μg) was labeled using the FlashTag™ Biotin RNA Labeling kit (Genisphere, Hatfield, PA) according to the supplier’s manual. The RNA of spike-in controls (oligos 2, 23, 29, 31, 36) were processed along with each sample RNA. The biotin-labeled RNA was hybridized on GeneChip® miRNA Arrays (Affymetrix). The wash and stain procedures were performed according to Genisphere’s suggestions on Affymetrix protocols (GeneChip® Expression Wash, Stain and ScanUser Manual, Affymetrix). The arrays were processed using the automated washing and staining protocol specified in the Fluidics Script FS450_03 (Affymetrix). After washing, the array was scanned using the GeneChip Scanner 3000 7G and the raw image data were saved in DAT files. Command Console Software (Affymetrix) was used to automatically grid the DAT files and to create the CEL files (probe cell intensity data).

### Microarray normalization

Normalization of the Exiqon raw data was performed in various ways, as detailed in Additional file 1: Table S1, always following the basic “background correction → normalization → summarization” structure inherited from RMA [36]. The LOWESS normalization was performed by Exiqon and consisted of a *normexp* background correction, a within-array *lowess* normalization and a median summarization [24]. The other approaches were implemented in both *R* and *Matlab* environments using standard functions available in the Bioconductor *limma* and *vsn* packages, and in the *Matlab* Bioinformatics toolbox. Normalization of the Affymetrix raw data was performed either using the default workflow of the Affymetrix software *miRNAQCtool* (http://www.affymetrix.com/Auth/products_services/software/download/mirnaqc/mirnaqc_terms.affx?v=Release_1_1_1) or, in the *R* environment, using functions from the Bioconductor packages *affy* and *makecdfenv*. These raw datasets and normalized datasets have been deposited in the ArrayExpress database (http://www.ebi.ac.uk/arrayexpress/) under accession numbers E-MTAB-876 (Exiqon miRCURY LNA™ platform, lung, and blood samples) and E-MTAB-875 (Affymetrix GeneChip® miRNA platform, lung, and blood samples).

### Spike-in controls based normalization method

This normalization method was developed to take into account the specificities of a miRNA array (compared with an mRNA array) and to exploit the information captured by the spike-in control probes.

The raw data are represented by a matrix *E*_*ik*_ containing the log2 of the measured intensities (“Intensity” column from an Affymetrix CEL file and “Signal Median” column from an ImaGene text file) and are labeled by the probe index *i* and the sample index *k*. The sub-matrix *E*^*(S)*^_*ik*_ containing only the spike-in control probes is considered first. After median summarization the matrix *E*^*(S)*^_*jk*_ is obtained, and labeled by the spike-in control probe set index *j*.

Ideally, all the columns of *E*^*(S)*^_*jk*_ should be equal, because the concentrations of the spiked-in RNA molecules *j* are the same across all samples *k*. In practice, this is rarely the case and correcting for these deviations is the goal of the normalization method described here. These relationships can be expressed by the general equation:

$${E^{\left(S\right)}}_{\mathrm{jk}}=E^{(S,}{{}^{\mathrm{mean})}}_{j}+\Delta {E^{\left(S\right)}}_{\mathrm{jk}}+\epsilon_{\mathrm{jk}},$$

where *E*^*(S,*mean*)*^_*j*_ is the mean of *E*^*(S)*^_*jk*_ over all samples *k*. Thus, *E*^*(S,*mean*)*^_*j*_ is the estimate of the actual spike-in control probe set intensities, independent of the sample *k*. The normalization corrections for the spike-in control probe set intensity values are captured by *ΔE*^*(S)*^_*jk*_ and *ϵ*_*jk*_ contains the residual errors. The goal is to construct a correction function *ΔE* based on *ΔE*^*(S)*^_*jk*_ that can be applied subsequently to all values of *E*_*jk*_. It is assumed here that the deviations that affect the spike-in control probes are the same as the deviations that affect the experimental miRNA probes (assumption A0).

The main factors expected to affect *ΔE*^*(S)*^_*jk*_ are the sample *k*, the measured intensity *E*^*(S)*^_*jk*_, and the channel color (when applicable). These dependences are implemented in the correction function *ΔE* based on the correlation matrix *C*_*jj’*_ of the rows of the spike-in control probe set intensities matrix *E*^*(S)*^_*jk*_. If the off-diagonal values in *C*_*jj’*_ are all close to 0 (below 0.5), this means that the differences *E*^*(S)*^_*jk*_ - *E*^*(S,*mean*)*^_*j*_ have different dependences in *k* for different *j*. In this case, it is not possible to extract a *k*-dependence common to all the spike-in control probe sets *j*, which would capture the common sample-specific deviations that need to be corrected by the normalization process. If, on the other hand, the off-diagonal values in *C*_*jj’*_ are all reasonably close to 1 (above 0.5), this means that the differences *E*^*(S)*^_*jk*_ - *E*^*(S,*mean*)*^_*j*_ display significant similarities in their dependences in *k* for different *j*. In other words, a substantial part of the individual variance of any spike-in control probe set *j* is “shared” with other probe sets *j’ ≠ j* (assumption A1). In this case, the observed coherence of the intensities of the spike-in control probe sets allows the separation of the signal *ΔE*^*(S)*^_*jk*_ from the noise *ϵ*_*jk*_ in the differences *E*^*(S)*^_*jk*_ - *E*^*(S,*mean*)*^_*j*_ (see *First step* below). The resulting correction function *ΔE* is then appropriate for performing a multi-array and intensity-dependent normalization of the raw data *E*_*ik*_. Compared with the single-array approaches, such as calculating a LOWESS curve from the spike-in control probe intensities *E*^*(S)*^_*jk*_ for each array *k*, the approach outlined above is expected to be more robust because of its multi-array characteristic. This characteristic will be particularly important for guaranteeing a stable extrapolation at high intensities where the coverage by the spike-in controls can be quite sparse (see *Second step* below, and Figure 1D).

#### First step

The first step in constructing the correction function *ΔE* is to calculate the normalization corrections *ΔE*^*(S)*^_*ik*_ for the spike-in control probe sets. Following the approach used to compute the correlation matrix *C*_*jj’*_, a centering and norming transformation is first applied to every spike-in control probe set intensity *E*^*(S)*^_*jk*_:

$$\begin{array}{l} {E^{\left(S\right)}}_{\mathrm{jk}}\to {E^{\left(S\right)}}_{\mathrm{jk}}-E^{(S,}{{}^{\mathrm{mean})}}_{j}\\ \;\to U_{\mathrm{jk}}\equiv \left({E^{\left(S\right)}}_{\mathrm{jk}}-E^{(S,}{{}^{\mathrm{mean})}}_{j}\right)/\left|{E^{\left(S\right)}}_{\mathrm{jk}}-E^{(S,}{{}^{\mathrm{mean})}}_{j}\right| \end{array}$$

The inclusion of an average taken over all samples *k* in the above transformation shows the multi-array nature of this method. Then *U*^(mean)^_*k*_ is computed as the mean over all the spike-in control probe sets *j* of *U*_*jk*_:

$$U_{\mathrm{jk}}\to {U^{\left(\mathrm{mean}\right)}}_{k}.$$

This step actually defines the “shared” variance present in the correlation matrix *C*_*jj’*_, which is purposely the same for all the spike-in control probe sets *j. U*^*(*mean*)*^_*k*_ is then transformed back to the original scale using the inverse of the above centering-norming transformation defined for each spike-in control probe set *j* and sample *k* as:

$$U^{(}{{}^{\mathrm{mean})}}_{k}\to \Delta {E^{\left(S\right)}}_{\mathrm{jk}}\equiv \left|{E^{\left(S\right)}}_{\mathrm{jk}}-E^{(S,}{{}^{\mathrm{mean})}}_{j}\right|*U^{(}{{}^{\mathrm{mean})}}_{k}.$$

This equation defines the normalization corrections for the spike-in control probe set intensity values *ΔE*^*(S)*^_*jk*_. The transformation of *U*^*(*mean*)*^_*k*_ introduces the intensity dependence of the correction because the scaling factor |*E*^*(S)*^_*jk*_*- E*^*(S,*mean*)*^_*j*_| depends on the spike-in control probe set *j* and thereby on the corresponding intensity range. The extension *ΔE*^*(S)*^_*ik*_ to the corresponding spike-in control probes *i* is performed simply by assigning the value of the corresponding probe set *j* to each probe *i*. The choice of performing the correction at the probe set level (*j*) rather than at the probe level (*i*) is expected to bring more stability in constructing the correction function *ΔE* and to allow clearer displays. However, constructing *ΔE*^*(S)*^_*ik*_ exclusively at the probe level would also have been possible and would have produced similar results.

#### Second step

The second step in constructing the correction function *ΔE* is to determine its value on all probe intensity values from the raw data matrix *E*_*ik*_. Keeping the discrete sample (*k*) dependence for *ΔE* and extending the spike-in control probe (*i*) dependence in a continuous intensity (*x*) dependence yields:

$$\begin{array}{l} \Delta {E^{\left(S\right)}}_{\mathrm{jk}}\mathrm{defined}\;\mathrm{for}\;\mathrm{the}\;\mathrm{intensity}\;\mathrm{values}\;\mathrm{in}\;{E^{\left(S\right)}}_{\mathrm{ik}}\\ \to \mathrm{\Delta E}\left(x,k\right)\;\mathrm{defined}\;\mathrm{for}\;\mathrm{all}\;x\;\mathrm{in}\;E_{\mathrm{ik}}. \end{array}$$

Because spike-in control probes are expected to map to well-defined intensity ranges, this assumption appears reasonable for the current technologies (assumption A2). However, it is important to confirm that this is indeed the case. Typically, this is done by checking in *E*^*(S,*mean*)*^_*j*_ that the intensity standard deviations of single spike-in control probe sets are smaller than the intensity difference between distinct spike-in control probe sets. A further important element for the above extension is the coverage of the intensity range contained in the raw data matrix *E*_*ik*_, by the intensities provided by the limited set of spiked-in control RNA transcripts (assumption A3). If this coverage is too sparse, interpolations and extrapolations based on the values contained in *ΔE*^*(S)*^_*ik*_ might become critical. If the coverage is satisfactory, then this extension step can be performed safely. Even a limited extrapolation is possible, as long as the uncertainties on the extrapolated values lie within the values observed on the reference spike-in control probe sets. The following strategy is used for the extension step:

● Stabilization at low intensities (if needed): the intensity values in *E*^*(S)*^_*ik*_ are completed by the minimum of *E*_*ik*_, with a *ΔE* value given by the closest spike-in control probe.

● Linear extrapolation at high intensities (if needed): the intensity values in *E*^*(S)*^_*ik*_ are completed by the integer values from the interval between the maximum of *E*^*(S)*^_*ik*_ and the maximum of *E*_*ik*_. The corresponding *ΔE* values are obtained from a linear regression based on the *ΔE*^*(S)*^_*ik*_ values of the two spike-in control probes with the highest intensities in *E*^*(S)*^_*jk*_.

● Smoothing the data: the (possibly extended) *E*^*(S)*^_*ik*_ and *ΔE*^*(S)*^_*ik*_ values are smoothed using a LOWESS procedure with a smoothing parameter of 0.4.

● Interpolation: the resulting transformed *E*^*(S)*^_*ik*_ and *ΔE*^*(S,*smooth*)*^_*ik*_) values are used in a simple interpolation procedure to generate *ΔE*_*ik*_, the values of *ΔE* for all elements of *E*_*ik*_.

Once the correction values *ΔE*_*ik*_ are available, the normalized data can be computed readily as:

$$E_{\mathrm{ik}}\to E^{(}{{}^{\mathrm{corr})}}_{\mathrm{ik}}\equiv E_{\mathrm{ik}}-\Delta E_{\mathrm{ik}}.$$

### Determination of the miRNA probe set detection calls

The Affymetrix *miRNAQCtool* software compares the four probes testing a given miRNA with the corresponding background probes, which are selected on the basis of an identical sequence length and identical GC-nucleotide content. The Wilcoxon test is performed to compare the two intensity distributions and the detection call is “present” when the resulting p-value is smaller than 0.06.

Exiqon defines a miRNA probe set as “present” if at least two of the four corresponding probes satisfy two conditions. First, their measured signal is not flagged as “bad quality” by the ImaGene scanning software. Second, their value must be larger than 1.5 times the median of all measured probe intensities on the array.

### Coefficients of variation

The “within-array” coefficient of variation measures the dispersion of the raw intensities *E*_*ik*_ between all the probes *i* corresponding to the probe set *j* in sample *k* as:

$$\mathrm{CVwithi}n_{\mathrm{jk}}=\frac{\mathrm{std}\;\mathrm{de}v_{\mathrm{probe}\;i\;\mathrm{mapping}\;\mathrm{to}\;\mathrm{probeset}\;j}\;E_{\mathrm{ik}}}{\mathrm{mea}n_{\mathrm{probe}\;i\;\mathrm{mapping}\;\mathrm{to}\;\mathrm{probeset}\;j}\;E_{\mathrm{ik}}}$$

The “between-array” coefficient of variation measures the dispersion of the normalized intensities *E*_*jk*_ of the probe set *j* between all the samples *k* belonging to the same sample group *l* (i.e., the samples *k* are biological replicates) as:

$$\mathrm{CVbetwee}n_{\mathrm{jl}}=\frac{\mathrm{std}\;\mathrm{de}v_{\mathrm{samples}\;k\;\mathrm{belonging}\;\mathrm{to}\;\mathrm{sample}\;\mathrm{group}\;l}\;E_{\mathrm{jk}}}{\mathrm{mea}n_{\mathrm{samples}\;k\;\mathrm{belonging}\;\mathrm{to}\;\mathrm{sample}\;\mathrm{group}\;l}\;E_{\mathrm{jk}}}$$

The *CVbetween* values of all the sample groups *l* are used to generate the distributions displayed in the boxplots in some of the figures.

The “treatment-induced coefficient of variation” extends the above measures of dispersion to the differential expression values obtained by comparing treated sample groups *l* with the control sample group. For a sample *k* in sample group *l* of size *N*_*l*_, the “residual variation” of the probe set *j* is taken as *R*_*jk*_ = *E*_*jk*_ – mean _samples *m* belonging to sample group *l*_*E*_*jm*_. The “treatment-induced coefficient of variation” is then defined by the following equation (similar to the inverse of the *t* statistic associated to the treatment *l* vs. control pairwise comparison):

$$\mathrm{CVtrea}t_{\mathrm{jl}}=\frac{\frac{1}{N_{l}+N_{\mathrm{control}}}\;\mathrm{sqrt}(\sum_{\mathrm{samples}\;k\;\mathrm{belonging}\;\mathrm{to}\;\mathrm{sample}\;\mathrm{group}\;l\;\mathrm{or}\;\mathrm{to}\;\mathrm{control}\;\mathrm{group}}R_{\mathrm{jk}}^{2})}{\mathrm{mea}n_{\mathrm{samples}\;k\;\mathrm{belonging}\;\mathrm{to}\;\mathrm{sample}\;\mathrm{group}\;l}\;E_{\mathrm{jk}}-\mathrm{mea}n_{\mathrm{samples}\;k\;\mathrm{belonging}\;\mathrm{to}\;\mathrm{control}\;\mathrm{group}}E_{\mathrm{jk}}}$$

The *CVtreat* values of all the sample groups *l* ≠ control are used to generate the distributions displayed in the boxplots in some of the figures.

The “spike-in” coefficient of variation measures the dispersion of the normalized intensities *E*^*(S)*^_*jk*_ of the spike-in control probe set *j* between all samples *k* in the dataset (in this context the samples *k* are technical replicates) as:

$$\mathrm{CVspik}e_{j}=\frac{\mathrm{std}\;\mathrm{de}v_{\mathrm{samples}\;k}\;{E^{\left(S\right)}}_{\mathrm{jk}}}{\mathrm{mea}n_{\mathrm{samples}\;k}\;{E^{\left(S\right)}}_{\mathrm{jk}}}$$

The “relative spike-in” coefficient of variation *relativeCVspike* is obtained by applying *CVspike* values to the above the linear transformation that maps the range of the *CVbetween* distribution to the interval [0,1]. The range of the *CVbetween* distribution is taken as the two extreme values within the interval [Q1-1.5*(Q3-Q1), Q3 + 1.5*(Q3-Q1)], where Q1 and Q3 are the first and third quartiles. This interval corresponds to the black whiskers in the boxplots used in the figures. Because it is not necessary equal to the interval given by the smallest and the largest values of the *CVbetween* distributions, *relativeCVspike* may have values outside the [0,1] interval.

### Calculation of treatment-induced differential miRNA expression

Given the matrix *E*_*jk*_ of normalized expression defined over all samples *k* and the corresponding normalized dose vector *D*_*k*_, a simple linear model is used to determine the treatment-induced differential expression for each probe set *j*. The corresponding (moderated) t-statistics were calculated with the Bioconductor *limma* package [24].

### Reverse transcriptase quantitative PCR (RT-qPCR)

Applied Biosystems TaqMan® miRNA assays were used for RT-qPCR. Because of its very high sensitivity (only 1–10 ng total RNA is required) and specificity, RT-qPCR is commonly used to confirm the results obtained with other platforms. TaqMan® MiRNA Assays use a stem-looped primer for reverse transcription and a sequence-specific TaqMan® assay. miRNA-specific cDNA was generated by reverse transcription with a Thermal Cycler (Applied Biosystems, Foster City, CA). The qPCR reaction and signal detection were performed with an Applied Biosystems 7900HT instrument according to the manufacturer’s instructions. The relative expression of the miRNAs was determined by the comparative *Ct*-method. First, the average of the *Ct* values, which were determined in triplicate, were calculated and the *ΔCt* values were calculated as *ΔCt* = *Ct*(target miRNA) – *Ct*(endogenous control; i.e., sno135). The *ΔCT* data were used to calculate the relative miRNA differential expression in the samples from the smoke-exposed animals as -*ΔΔCT* = *ΔCT*(“high”) – *ΔCT*(“sham”). The following Applied Biosystems TaqMan® miRNA assays were used: mmu-miR-146a (ID 000468), mmu-miR-342-3p (ID 002260), mmu-let-7i (ID 002221), mmu-miR-31 (ID 000185), mmu-miR-221 (ID 000524), mmu-miR-135b (ID 002261), mmu-miR-147 (ID 002262), mmu-miR-146b (ID 002453), mmu-miR-21 (ID 002493), mmu-miR-144 (197375_mat), mmu-let-7b (ID 002619), mmu-let-7c (ID 000379), mmu-miR-30a (ID 000417), mmu-miR-26a (ID 000405), mmu-let-7a (ID 002478), mmu-miR-30c (ID 000419), mmu-let-7f (ID 000382), mmu-let-7d (ID 001178), mmu-let-7 g (ID 002492), mmu-miR-34c (ID 002584), mmu-miR-99a (ID 000435), mmu-miR-195 (ID 000494), mmu-miR-125b-5p (ID 000449), and mmu-miR-99b (ID 000436). snoRNA135 (ID 001230) was used as the internal control RNA. Pre-experiments revealed that the linear range of the RT-qPCR was at 5 ng RNA per reaction. However, for one assay (mmu-miR-99b), 2.5 ng RNA was determined to be in the linear range of the RT-qPCR.

## Competing interests

The authors declare that they have no competing financial or other interests in relation to the work described in this manuscript.

## Authors’ contributions

AS developed and applied the methodology, led its implementation in *R*, and wrote the manuscript. SG wrote the *ExiMiR* package. UK, EV, and WH supervised the generation of the sample and raw data. AH supervised the generation of the raw data and wrote the biological interpretation. JH and MCP developed the overarching strategy and supervised the project. All authors read and approved the final manuscript.

## Supplementary Material

Additional file 1 **Table S1.** miRNA raw data preprocessing pipelines used in this study. Click here for file

Additional file 2 **Supplementary results. Figure S1.** Comparison of the raw intensities and of the “present”/”absent” calls. **Figure S2.** Inapplicability of the spike-in control based normalization for the Affymetrix lung dataset. **Figure S3.** Quality control metrics for the lung samples and all preprocessing pipelines. **Figure S4.** Quality control metrics for the blood samples and all preprocessing pipelines. **Figure S5.** Differential miRNA expression for the lung samples and all preprocessing pipelines. Click here for file
