# Supplementary material for: Assessment of a novel multi-array normalization method based on spike-in control probes suitable for microRNA datasets with global decreases in expression
Source: BMC Res Notes. 2014 May 17;7:302. doi: 10.1186/1756-0500-7-302 (PMC4077261; doi:10.1186/1756-0500-7-302)
Supplement: Additional file 1 — Table S1. miRNA raw data preprocessing pipelines used in this study. [file 1756-0500-7-302-S1.pdf]

ID	Pipeline	Raw data acquisition technology	Raw data type	Computational Environment	Background subtraction[a]	Normalization (*Bioconductor package*)	Summarization	Reference
**SCN**	Spike-in controls based	Exiqon miRCURY LNA™ array v11	ImaGene TXT files	R/Matlab	skipped	*ExiMiR*	Median	This work.
**SCVSN**	Spike-in controls combined with VSN					VSN on spike-in controls (*vsn*)		[16]
**MEDIAN**	Median normalization					Subtraction of array medians		
**LOWESS**	Exiqon LOWESS normalization			Performed by Exiqon	*normexp (limma)*	Within-array LOWESS (*limma*)		[35]
**VSN**	Variance stabilization			R/Matlab	skipped	*vsn*		[24]
**QN**	Quantile normalization					Quantile (*limma*)		[35]
**QNm**	Quantile normalization (mouse probes					Quantile on mouse probes (*limma*)		
**IS**	Invariant Set					Invariant set (*affy*)		[36]
**LVS**	Least-Variant Set					*LVSmiRNA*		[17]
**LRIN**	LUMI: rank-invariant					rankinvariant (*lumi*)	Median (performed before normliaztion)	[26]
**LVSN**	LUMI: variance stabilization					vsn (*lumi*)		
**LRS**	LUMI:roubst splines					rin (*lumi*)		
**NN**	No normalization					Skipped	Median	
**AQN**	Affymetrix quantile normalization	Affymetrix GeneChip® miRNA array	CEL files	Windows XP	Default workflow in *miRNAQCtool* v.1.0			

ORIGINAL VERSION (from submitted manuscript)

ID	Pipeline	Raw data acquisition technology	Raw data type	Computational Environment	Background subtraction[a]	Normalization (*Bioconductor package*)	Summarization	Reference
**SCN**	Spike-in controls based	Exiqon miRCURY LNA™ array v11	ImaGene TXT files	R/Matlab	skipped	*ExiMiR*	Median	This work.
**SCVSN**	Spike-in controls combined with VSN	“	“	“	“	VSN on spike-in controls (*vsn*)	“	[16]
**MEDIAN**	Median normalization	“	“	“	“	Subtraction of array medians	“	
**LOWESS**	Exiqon LOWESS normalization	“	“	Performed by Exiqon	*normexp (limma)*	Within-array LOWESS (*limma*)	“	[35]
**VSN**	Variance stabilization	“	“	R/Matlab	skipped	*vsn*	“	[24]
**QN**	Quantile normalization	“	“	“	“	Quantile (*limma*)	“	[35]
**QNm**	Quantile normalization (mouse probes	“	“	“	“	Quantile on mouse probes (*limma*)	“	“
**IS**	Invariant Set	“	“	“	“	Invariant set (*affy*)	“	[36]
**LVS**	Least-Variant Set	“	“	“	“	*LVSmiRNA*	“	[17]
**LRIN**	LUMI: rank-invariant	“	“	“	“	rankinvariant (*lumi*)	Median (performed before normliaztion)	[26]
**LVSN**	LUMI: variance stabilization	“	“	“	“	vsn (*lumi*)		“
**LRS**	LUMI:roubst splines	“	“	“	“	rin (*lumi*)		“
**NN**	No normalization	“	“	“	“	Skipped	Median	
**AQN**	Affymetrix quantile normalization	Affymetrix GeneChip® miRNA array	CEL files	Windows XP	Default workflow in *miRNAQCtool* v.1.0			

CORRECTED VERSION (with *same as above* symbol “ in empty cells)
