# Supplementary material for: Assessment of a novel multi-array normalization method based on spike-in control probes suitable for microRNA datasets with global decreases in expression
Source: BMC Res Notes. 2014 May 17;7:302. doi: 10.1186/1756-0500-7-302 (PMC4077261; doi:10.1186/1756-0500-7-302)
Supplement: Additional file 2 — Supplementary results. Figure S1. Comparison of the raw intensities and of the “present”/”absent” calls. Figure S2. Inapplicability of the spike-in control based normalization for the Affymetrix lung dataset. Figure S3. Quality control metrics for the lung samples and all preprocessing pipelines. Figure S4. Quality control metrics for the blood samples and all preprocessing pipelines. Figure S5. Differential miRNA expression for the lung samples and all preprocessing pipelines. [file 1756-0500-7-302-S2.pdf]

Additional File

Supplementary Results

Comparison of raw data and detection calls obtained on the Exiqon and Affymetrix platforms

Raw data and detection calls obtained on the dual-channel platform were benchmarked against the same quantities obtained on the single-channel Affymetrix platform to assess the data quality prior to the normalization step. First, the raw probe intensities were considered taking each array separately. The analysis was restricted to the 2,380 mouse miRNA probes that were present on both platforms, which correspond to the 595 four-fold replicated common miRNA probe sets. These common probes constituted a subset of the 2,400 mouse probes (and the 9,360 total probes) present on the Exiqon miRCURY array, and of the 2,436 mouse probes (and the 46,227 total probes) present on the Affymetrix GeneChip® array. Figure S1A and S1B shows the distributions of the log2-transformed raw signal for the lung dataset from the Hy3 Exiqon miRCURY LNA™ and Affymetrix GeneChip® miRNA arrays, respectively. In both cases, the distributions cover a range of approximately nine and peak strongly at low values: 75% of the Exiqon values lie in the lower ~22% of the intensity range, and 75% of the Affymetrix values lie in the lower ~11% of the intensity range. This suggests that a majority of mouse miRNAs were weakly expressed in these samples and that the raw signal distribution was broader on the Exiqon platform compared with its distribution on the Affymetrix platform, indicating a higher sensitivity of the Exiqon dual-channel hybridization. The miRNA probe set detection calls measure the difference between the detected probe intensities and the background noise. Although the values are presented associated with normalized expression values in Figure S1C and S1D, they actually depend only on the raw data (see "Methods"). Therefore, only one representative normalization method was needed for each platform:

spike-in controls based normalization (SCN) for Exiqon and quantile normalization (AQN) for Affymetrix (see Table 1). Figure S1C shows the overall distributions of the miRNA probe set normalized intensities, split according to the associated “absent” and “present” detection call values. The intensity distributions shared similar features in the data from the two arrays. One of the features is a clear peak at the lower end of the detection range, which contained the “absent” miRNA probe sets. A second feature is that the distribution of the “present” probe sets was close to a straight line on the log-scaled intensity histogram, corresponding to a power law dependence in the absolute intensity scale. The slope of the Exiqon intensity data was slightly less steep than that of the Affymetrix data. Figure S1D shows an overall MA-plot between the SCN and AQN normalized datasets. Each common mouse miRNA probe set is colored according to its detection calls values on each platform. The asymmetry between the two sides of the horizontal black line indicates that the situation was different, depending on whether SCN or AQN produced the largest normalized intensity; that is, when the SCN intensity value was higher (upper part of the plot), the difference was greater than when the AQN intensity value higher (lower part of the plot). This feature also appeared in the detection calls comparison: the extension of the blue points (SCN:present-AQN:absent, 10% of the cases) was greater than the extension of the red points (SCN:absent-AQN:present, 16% of the cases). This finding suggests that, on average, the “pseudo-single-channel” hybridization strategy used on the Exiqon platform allowed more signal to be detected than were detected on the single-channel Affymetrix platform.

Supplementary Figures

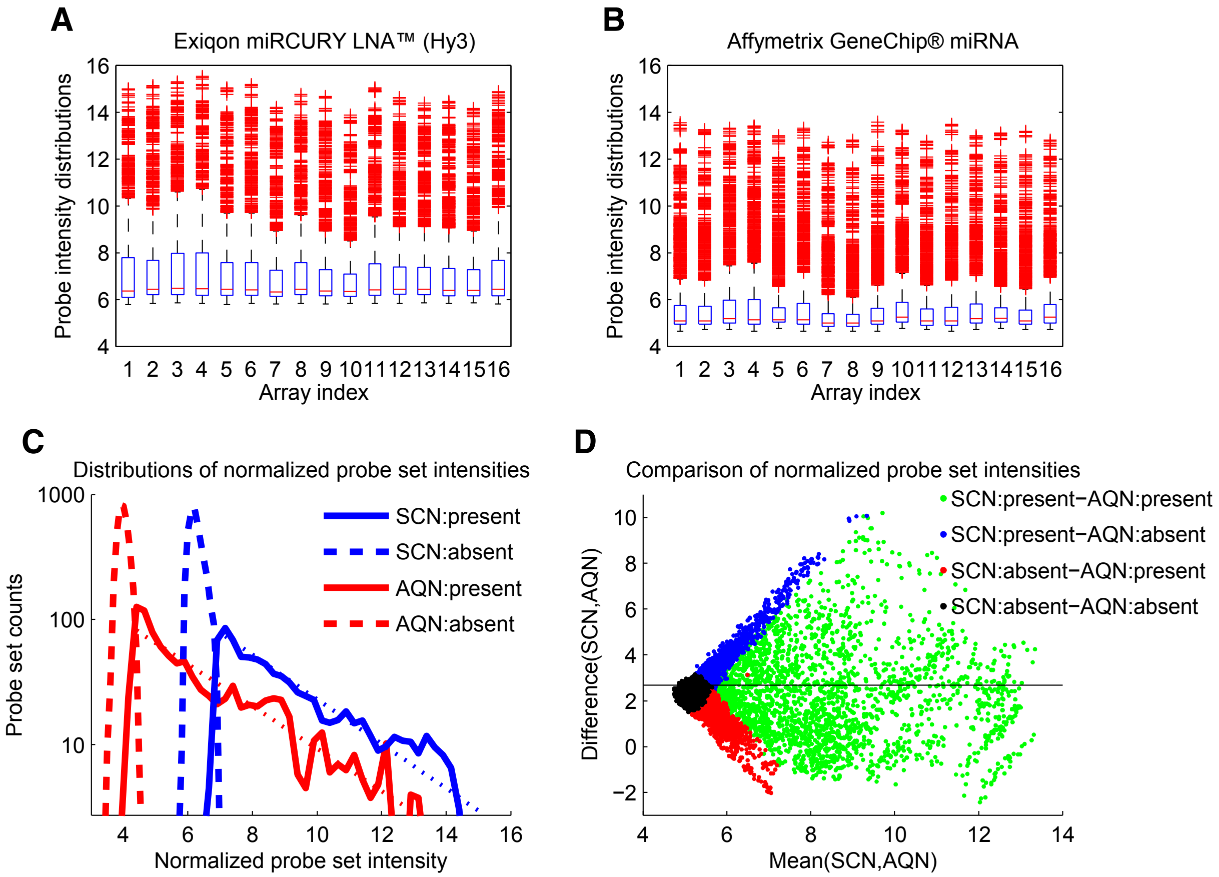

Figure S1. Comparison of the raw intensities and of the "present"/"absent" calls. (**A**, **B**) Boxplots of the log2 raw signal intensities of the 2,380 common mouse probes between the Hy3 Exiqon miRCURY LNA™ array (panel A) and the Affymetrix GeneChip® miRNA array (panel B). Median, red line; first and third quartiles Q1 and Q3, blue box; distribution range given by the two extreme values within the interval [Q1−1.5×(Q3−Q1), Q3+1.5×(Q3−Q1)], black whiskers; outliers, red crosses outside the whiskers. (**C**) Distribution of the normalized intensities for the "absent" and "present" miRNA probe sets.

(**D**) Overall MA-plot of the normalized intensities for the SCN (Exiqon) versus AQN (Affymetrix) comparison, including the 585 common miRNA probe sets measured in all 16 lung arrays. The horizontal black line represents the difference of minimal detected intensities between SCN and AQN, also visible in panel C.

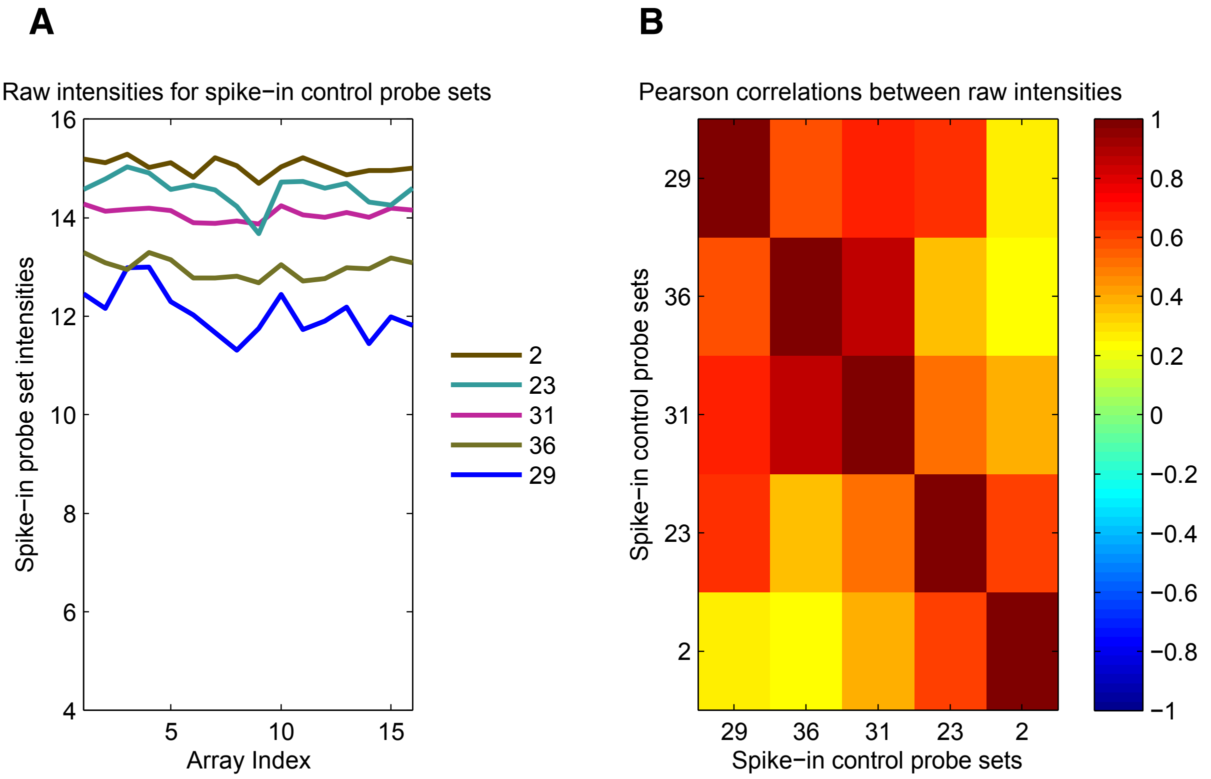

Figure S2. Inapplicability of the spike-in control based normalization for the Affymetrix lung dataset. (**A**) Raw intensities for the five spike-in control probe sets from the 16 arrays of the Affymetrix lung dataset. The probe set values were computed as the median values of the corresponding probes. (**B**) Heat map of the Pearson correlation matrix between all pairs of the spike-in control probe sets shown in panel A. These results show that assumption A1 was not satisfied because the mean of the off-diagonal Pearson correlations between the spike-in control probe sets was too low (0.51). Furthermore, both the intensity specificity and coverage of the spike-in probe sets were insufficient (assumptions A2 and A3, respectively). This led to the conclusion that the Affymetrix lung raw data were not suitable for the application of the spike-in controls based normalization method.

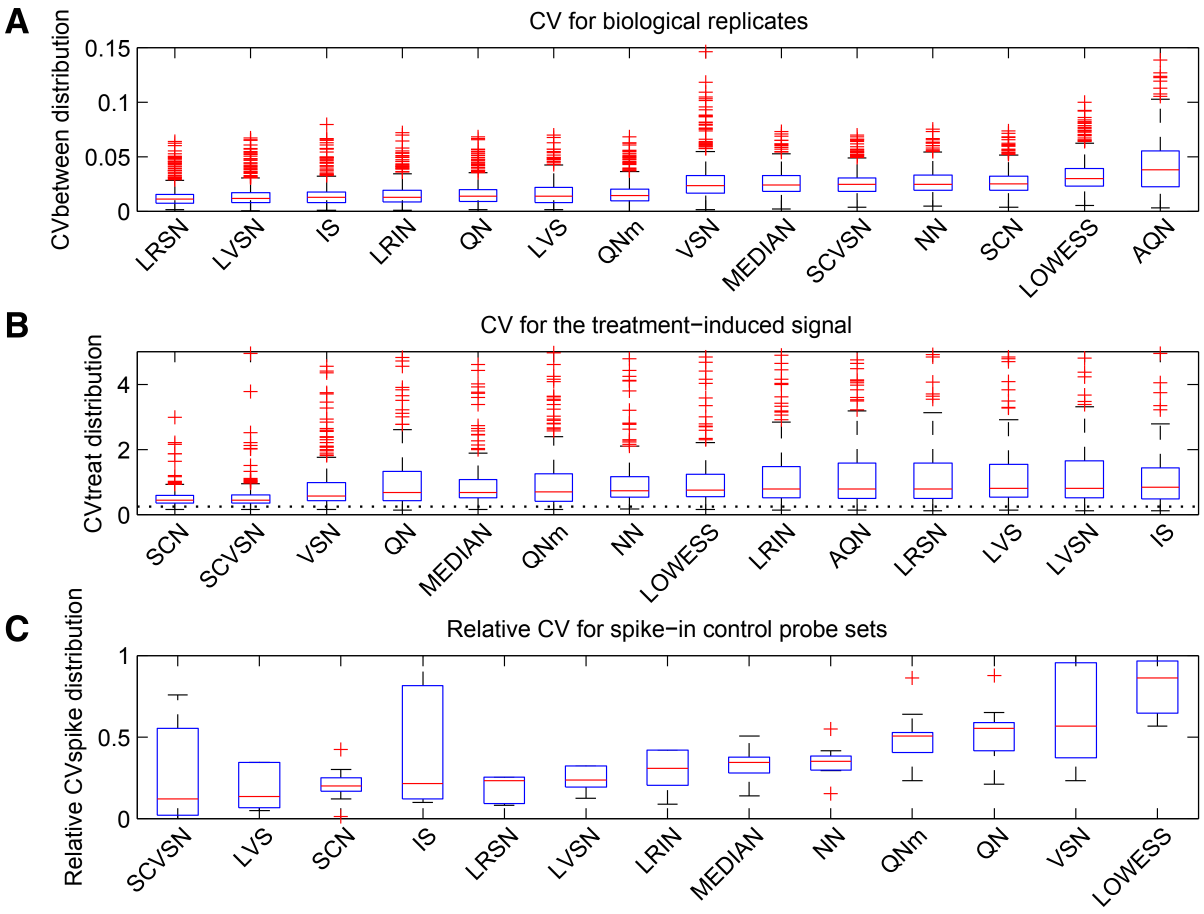

Figure S3. Quality control metrics for the lung samples and all preprocessing pipelines. (**A**) Boxplot of the CVs between the probe set normalized intensity values from the four biological replicates of a given treatment group (*CVbetween*), computed for all 595 common mouse miRNA probe sets and for all four treatment groups of the lung samples, excluding those with "absent" detection calls. (**B**) Boxplot of the ratio between the probe set residual variance and the corresponding modeled treatment response (*CVtreat*), computed for all 595 common mouse miRNA probe sets for the lung samples, excluding those with "absent" detection calls. (**C**) Boxplot of the CVs between the probe set normalized intensity values from all 16 arrays of the lung dataset, computed for the 10 Exiqon spike-in control probe sets. For each preprocessing pipeline (described in Table 1), the boxplot shows the values relative to the range of the corresponding *CVbetween* distribution, which is given by the whiskers in panel A and the interval [0,1] in panel C (*relativeCVspike* , see "Methods"). Median, red line; first and third quartiles Q1 and Q3, blue box;

distribution range given by the two extreme values within the interval [Q1−1.5×(Q3−Q1), Q3+1.5×(Q3−Q1)], black whiskers; outliers, red crosses outside the whiskers.

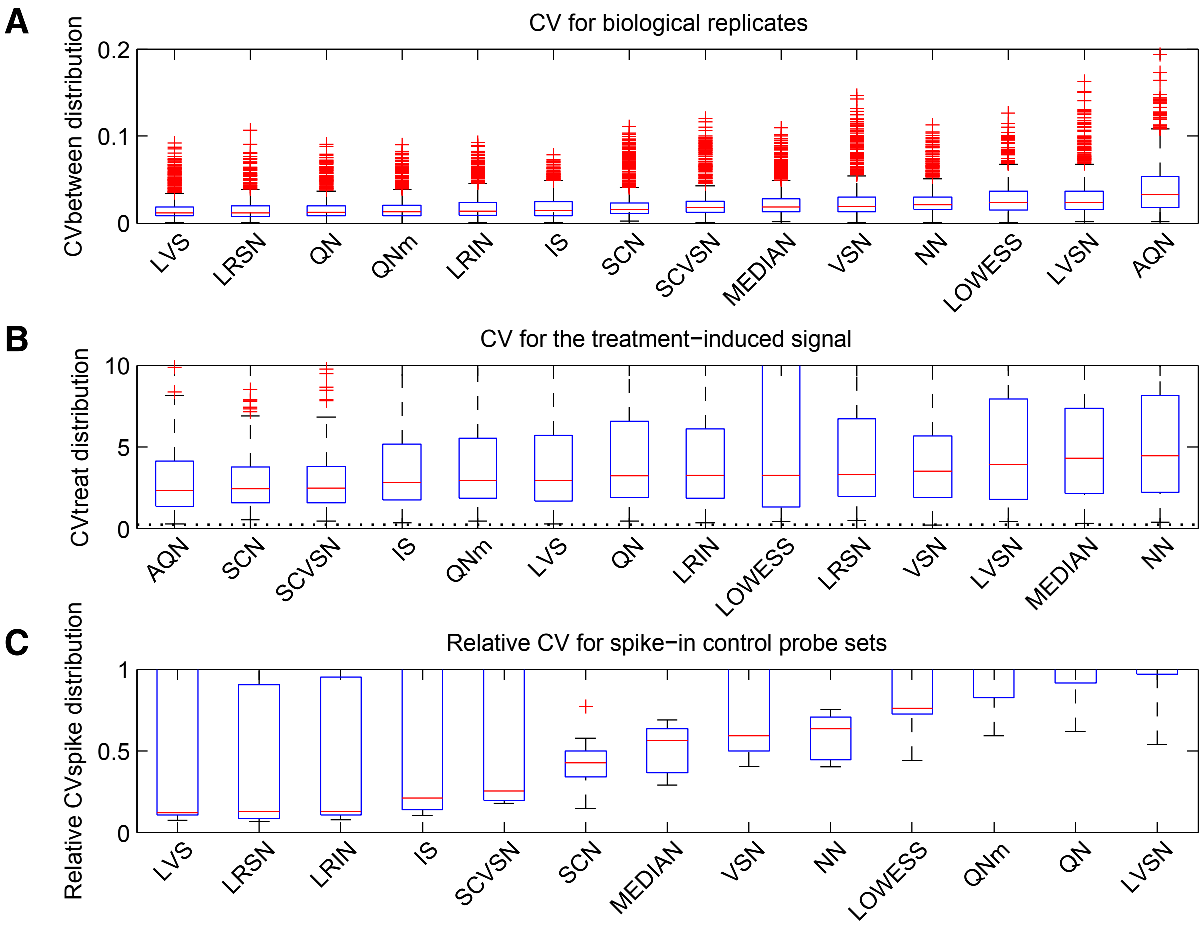

Figure S4. Quality control metrics for the blood samples and all preprocessing pipelines. (**A**) Boxplot of the CVs between the probe set normalized intensity values from the four biological replicates of a given treatment group (*CVbetween*), computed for all 595 common mouse miRNA probe sets and for all four treatment groups of the blood samples, excluding those with “absent” detection calls. (**B**) Boxplot of the ratio between the probe set residual variance and the corresponding modeled treatment response (*CVtreat*), computed for all 595 common mouse miRNA probe sets for the blood samples, excluding those with “absent” detection calls. (**C**) Boxplot of the CVs between the probe set normalized intensity values from all 16 arrays of the blood dataset, computed for the 10 Exiqon spike-in control probe sets. For each preprocessing pipeline (described in Table 1), the boxplot shows the values relative to the range of the corresponding *CVbetween* distribution, which is given by the whiskers in panel A and the interval [0,1] in panel C (*relativeCVspike* , see “Methods”). Median, red line; first and third quartiles Q1 and Q3, blue box;

distribution range given by the two extreme values within the interval [Q1−1.5×(Q3-Q1), Q3+1.5×(Q3−Q1)], black whiskers; outliers, red crosses outside the whiskers.

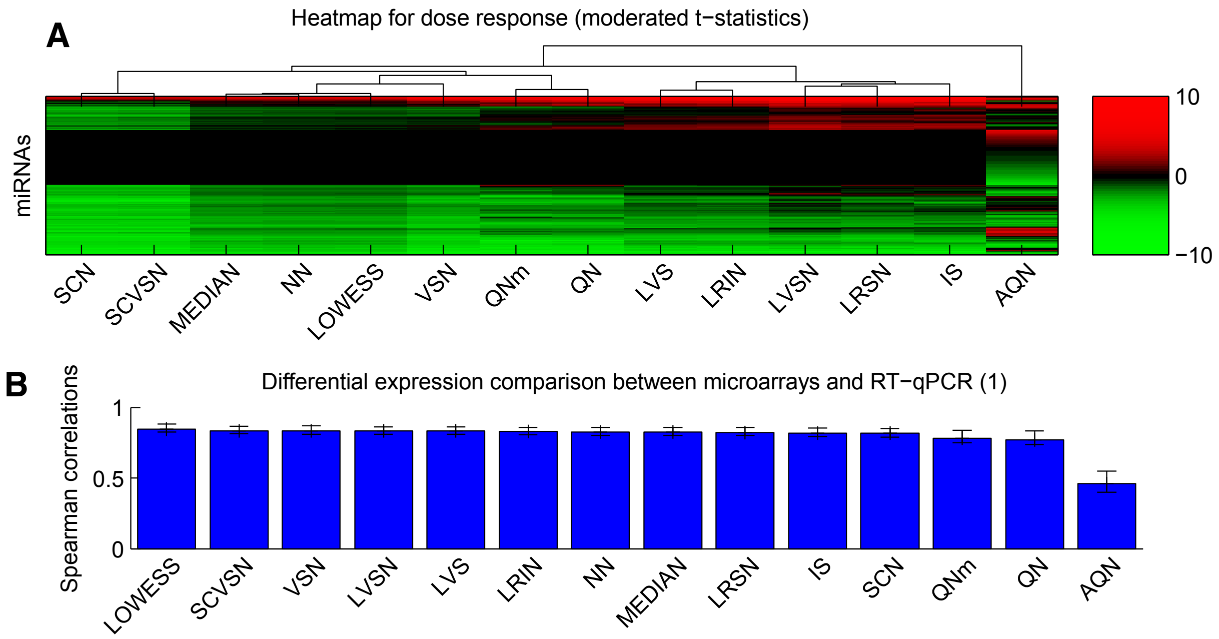

Figure S5. Differential miRNA expression for the lung samples and all preprocessing pipelines. (**A**) Heat map for t-statistics obtained from the linear model for the treatment response of the expression values of each miRNA. The dendrogram is based on the Euclidean distance between the t-values obtained from the various preprocessing pipelines (described in Table 1). (**B**) Bar chart of the Spearman correlation coefficients between the differential expressions of the selected miRNAs obtained by RT-qPCR and those obtained from the preprocessing pipelines. The error bars are the 2.5^{th}–97.5^{th} percentiles of the values obtained from a simple leave-one-out re-sampling approach.
